# Fellowships, Grants, & Awards

**Published:** 2007-06

## Global Network for Women’s and Children’s Health Research (U01)

The NICHD invites applications from investigators willing to participate under a cooperative agreement in an ongoing multicenter international research network designed to perform randomized clinical trials of interventions to reduce the major risks to maternal, neonatal, infant, and early childhood health in resource-poor countries. The purpose of this solicitation is to complement the existing Global Network with the addition of research units (RUs) from Africa and India. The objective of this program is to contribute to the resolution of maternal and pediatric health problems by establishing a network of RUs (paired U.S.-based and foreign centers) that will use common protocols to implement randomized clinical trials and thus contribute to the evidence base for sound clinical, programmatic, and policy decisions. The network will establish the infrastructure necessary to initiate, implement, and evaluate randomized controlled trials in community settings and health care facilities among pregnant women, newborns, infants, and children to the age of 3 years.

The overall goal of the network is to expand scientific knowledge relevant to improving health outcomes for women and children in developing countries. Other critical goals are to: 1) develop sustainable research infrastructure and public health intervention capabilities in developing countries and 2) strengthen international collaborative research arrangements that focus on the leading causes of morbidity and mortality in pregnancy and early childhood. A key objective for the network RUs is to design, develop, and conduct multiple simultaneous common clinical trials, as well as to evaluate and implement evidence-based health interventions and pertinent formative and translational research studies collaboratively. These studies must have a strong scientific and epidemiologic basis for their use in a foreign country, and should be culturally appropriate. The primary end points in these studies must be associated with demonstrable improvement in important public health measures in the population under study. This network will bring the required numbers of subjects into rigorously designed common protocols and thus address pressing research questions in pregnant women and very young children more quickly and efficiently than could individual centers acting alone.

Research that is predominantly epidemiologic, such as observational or surveillance studies, will not be reviewed and will be returned to the PI as unresponsive to the RFA. Applications that propose only to evaluate health care delivery programs or health care utilization and do not evaluate a human experimental intervention will also be considered as non-responsive and will be returned without review. Program staff will assist the PIs and the SFIs of the Global Network for Women's and Children's Health Research (GN) to identify research topics of high priority, and to design, implement, and evaluate the impact of common protocols.

According to a report of the Global Forum for Health Research, > 1.3 billion of the approximately 6 billion people alive today live in extreme poverty. Nearly 3 billion people live on < $2 a day, and 70% of these poor are women. Frequently in poor health, they live in unsafe and unsanitary conditions, have the primary responsibility for caring for children and the elderly, and are often expected to perform the major tasks of family sustenance. Despite successes during the past 30 years in improving the health of women and children worldwide, interventions are needed to avert the continuing unnecessary, preventable deaths, illness, and disability that disproportionately affect poor women and children, especially in the developing world.

Problems at the home and community level contribute to maternal, neonatal, and child morbidity and mortality. These include underlying nutritional deficits, limited access to quality medical care, inadequately trained health care workers, lack of adequate health information and resources, social and cultural norms, civil and domestic violence, and the low social status of women and children.

According to World Bank reports, maternal mortality remains the human development indicator that shows the greatest gap between developing and developed countries. The health problems that contribute most to maternal deaths in the developing world include intrapartum bleeding, sepsis, eclampsia, obstructed labor, and postpartum hemorrhage. The World Health Organization (WHO) estimates that every year > 500,000 women die as a result of pregnancy-related complications, most due to postpartum hemorrhage or infection. This represents one in 16 women in developing countries dying of pregnancy-related complications, compared with one in every 2,800 women in developed countries. Seventy percent of women receive no postpartum care in the 6 weeks following delivery.

For every woman who dies, an estimated 15–30 survive but suffer chronic disabilities and morbidities. This includes obstetric fistula, one of the most common, painful, and stigmatizing disabilities. Obstetric fistula affects approximately 2 million women, with 50,000–100,000 new cases per year.

Improving maternal health in the developing world is of utmost importance for the woman's own sake, and because the death of a reproductive age woman significantly lowers the chance of survival of her infants and children. The WHO estimates that approximately 8 million infants die each year, and of these, more than half (4.4 million) die within the first 28 days of life.

Ninety-eight percent of these deaths occur in developing countries and are caused by infectious diseases; pregnancy-related conditions; and delivery-related complications, including intrapartum asphyxia, birth trauma, and premature birth. Birth asphyxia accounts for 21% of deaths, followed by pneumonia (19%) and neonatal tetanus (14%). These deaths are preventable if women and their neonates have access to trained health care workers for delivery, immunizations, and timely treatment of infections.

An estimated 10 million children < 5 years of age, primarily in developing countries, die each year from infectious diseases, nutrition-related illnesses, and perinatal complications. These conditions, and others associated with early childhood, are expected to remain the major causes of mortality in young children through 2020. Two-thirds of these illnesses and deaths are preventable, yet they are associated with about half of the disease burden in the poorest regions of the world, including India and sub-Saharan Africa, where the rate of improvement in child mortality is actually slowing.

Infectious diseases account for 63% of deaths in children < 5 and many, such as tuberculosis and malaria, have become more difficult to treat due to increased resistance to commonly used and available antimicrobial drugs. The WHO estimates that 10,000 maternal deaths and 200,000 infant deaths each year in the developing world are associated with malaria infection during pregnancy. Lower immunity during pregnancy puts women at increased risk for malaria, which is associated with intrauterine growth retardation, increased fetal losses, susceptibility to other illnesses and death. Over 40% of the world's children live in malaria endemic areas. The WHO estimates that malaria causes approximately 20% of all childhood deaths in Africa. Despite increased resistance to antimalarial drugs, many of these deaths are still preventable with appropriate interventions.

Because > 40% of the world’s children live in malaria endemic areas, older children are also at risk. In developing countries, where an estimated 63% of infants are born at home and an estimated 80% of children die at home, advice and assistance concerning reproductive health, birth practices, and infant and child care may be available only from local community health workers, traditional medical practitioners, or members of a woman's extended family. Research is needed to identify interventions that can be delivered in community and primary care settings because referral and transport to higher level institutional care are often unavailable.

Toxic environmental exposures, including cooking smoke and tobacco exposure, represent an increasing threat to women. Women comprise > 200 million of the world's 1.2 billion cigarette smokers. Maternal cigarette smoking is associated with perinatal mortality, low birth weight, premature delivery, and other adverse outcomes, while environmental tobacco smoke exposure (ETS) poses a serious risk to infants and young children as well as to adults. Although female smoking prevalence is still quite low in many developing countries, it appears to be increasing, and women's use of other forms of tobacco is common in some regions. Research is needed on interventions to prevent and reduce tobacco use and on ETS exposure among pregnant and reproductive age women and girls in developing countries.

Given the alarming rates of morbidity and mortality in women and children in the developing world, randomized clinical trials testing innovative interventions are urgently needed to help improve the health of women and children in these countries. In some instances, the results of efficacy studies have not been translated into feasible interventions for resource-poor settings where the burden of these conditions is the heaviest. In other cases, feasible interventions have been developed, but they are relatively ineffective, or effectiveness has not been assessed. Because economic investment in early intervention is warranted, and in many situations is relatively inexpensive, rigorously tested health interventions can have enormous impact. A global network of U.S. and developing country scientist teams and institutions will facilitate high quality, sustainable, collaborative research that can address many of these problems and issues while simultaneously building the professional capacity and infrastructure in developing country sites.

Originally funded to perform site-specific diverse individual protocols, the GN has transitioned to the conduct of common research protocols to more efficiently build research capacity and address the major causes of morbidity and mortality among women and young children in the developing world. Grantees will be part of a GN that will include clinical RUs and a Data Coordinating Center. The GN is intended to strengthen and expand the global infrastructure for women's and children's health research. It will increase opportunities for scientific linkages, interaction, knowledge development and transfer, and collaborative partnerships among U.S. and foreign investigators and institutions.

This initiative calls for a broad array of interventional studies conducted across sites in the developing countries selected. The studies should address health conditions in women and young children, with an emphasis on the perinatal and neonatal periods, but up to and including 3 years of age. Proposed interventions should emphasize the development, testing, and adaptation of cost-effective, integrated biomedical, behavioral, social, and public health interventions that may reduce causes of premature morbidity and mortality among women of reproductive age and young children. Applications based predominantly in basic laboratories in the United States will not be considered responsive to the RFA. Studies focused on health interventions performed at the community and primary health care level are of particular interest to the GN.

The GN will function as an affiliated group that fosters communication, innovation, and research excellence. Applications for meritorious common research studies that are relevant to the health needs of more than one locality will be reviewed and funded under the network. Grantees should expect to propose and participate in multiple common protocols that may be conducted in several or all network sites. Grantees must be willing to work collaboratively and with cultural sensitivity.

All research conducted under the auspices of the GN must be designed such that health improvements in the study population are meaningful, sustainable, and likely to represent a measurable and significantly improved health outcome. Surrogate or intermediate outcomes must be well defined and scientifically well justified.

The GN emphasizes a multidisciplinary, team-based approach. Disciplines may include pediatrics, family medicine, obstetrics, infectious diseases, epidemiology, statistics, environmental science, pharmacology, and the behavioral and social sciences. GN teams should be based primarily at the institution of the SFI. It is not the intention of this RFA to support multidisciplinary teams of investigators in the United States.

Priority will be given to scientific activities that have the greatest likelihood of improving pregnancy and child outcomes in developing countries. Suitable topics for proposed research projects include, but are not limited to, the following: 1) prevention or reduction of leading causes of maternal morbidity and mortality, including but not limited to, malnutrition, infectious diseases, hypertensive disorders, obstructed labor, postpartum hemorrhage, cervical cancer, and cancers exacerbated by environmental exposures; 2) studies focused on assessing and improving pregnancy management, including reduction of infection, emergency care, birth practices, resuscitation, and postpartum care of the mother, neonate, and young infant; 3) prevention and reduction of fetal loss, stillbirths, preterm delivery, and birth defects; 4) prevention and treatment of infectious diseases in infants and young children up to 3 years, including, but not limited to, sepsis, malaria and other parasitic diseases, diarrheal diseases, acute respiratory tract infections, and vaccine-preventable infections; 5) within GN clinical trials, conduct research that describes major individual, social and cultural factors (such as belief systems, affordability, availability, and access) influencing health-seeking behavior; 6) nutritional interventions that enhance pregnancy outcomes and infant growth, development, energy, and immunity; 7) early interventions to enhance child outcomes, including parental caretaking and parental mental health; 8) prevention and treatment of environmental exposures in girls and women of reproductive age and young children up to 3 years, including, but not limited to, tobacco, indoor pollution, toxic chemicals, and water-borne infectious agents; 9) studies to evaluate cost-effective, new rapid diagnostic methodologies (e.g., tests for sepsis, malaria, tuberculosis, etc., and verbal autopsy) available to improve identification of maternal, infant, and young child morbidity, and/or mortality.

To provide an idea of the capabilities of the applicant RUs to participate in the development and design of cooperative protocols, each applicant should propose one fully developed common research study to address a current critical or emerging health problem related to the needs of women and/or young children in the foreign collaborator's country and other developing countries. The protocol should be appropriate for the GN in that it requires a multicenter design. The application should provide a research plan that includes an evidence-based statement of the health problem (including preliminary data); research hypothesis; specific aims; proposed study population; study design, methodology, and recruitment plans; data collection, analysis, and reporting plans; suggested timelines and staffing plans; plans for enhancing research capacity at the foreign sites; plans for sustaining the intervention after the funding period is over; and a proposed budget for the life of the project. Evidence of collaboration and multidisciplinary expertise should be provided. It is critically important that the proposed intervention be community or primary care facility-based and not place undue burden on the population or cause disruption of local facilities. The quality of the research plan will be an important focus of the peer review of the application. There is no guarantee that the proposed research plan will be accepted as a common protocol by the GN. However, funded RUs will be invited to submit the protocols from their application to the Protocol Review Subcommittee and Steering Committee for review and consideration. The Global Network Data Coordinating Center will assist in data collection and management, including statistical expertise, and research protocol management.

The NICHD expects that ongoing common protocols of the currently-funded GN may continue into the continuation grant period in existing centers. New common protocols may be developed before the start of the continuation. Research sites that join the network during the next award period (beginning approximately 1 April 2008) may participate in the protocols ongoing at that time, as appropriate.

The NICHD expects the network to initiate new common protocols in the first year. The topics of these protocols will be decided and prioritized cooperatively by the Global Network Steering Committee and implemented after internal NIH review.

This RFA solicits applications for pairs of U.S. and developing country research sites to form a research network to address the major causes of maternal, neonatal, infant, and early childhood morbidity and mortality. The grantees will form a cooperative network in scientific partnership with NICHD to conduct common randomized clinical trials evaluating interventions in resource-poor settings. The catchment populations for the sites will include a diversity of ethnic, linguistic, and cultural groups.

### Organizational Components

The GN consists of multiple RUa composed of multidisciplinary teams of collaborating U.S. scientists linked to investigators in developing countries as full and equal partners; a single Data Coordinating Center that will provide research support services and methodological and statistical expertise for the GN; and the NIH (represented by the NICHD Program Scientist and Staff Science Collaborators from NICHD.) The GN RUs may include additional consultants needed at specific time periods.

### Steering Committee

The steering committee will serve as the GN’s central point of communication; exchange of ideas; development, review, and management of common protocols; research and training activities; and problem resolution. The steering committee reviews all presentations and publications via the publications subcommittee and NICHD clearance mechanisms. The steering committee is composed of the U.S. PI and SFI of each RU, the PI of the Data Coordinating Center, and the NICHD Program Scientist, each of whom have one vote. NICHD will appoint a chairperson who is not participating as a PI for a minimum term of one year.

The steering committee will meet twice annually in person and will participate in telephone or video conferences as needed between steering committee meetings. During the meetings, research progress and problems are reviewed for each site.

### Data Coordinating Center

The GN Data Coordinating Center has the responsibility to provide the network's organizational, statistical, and technical support, including the shared funding of information technology staff and site training, and regulatory adherence. In addition, the Data Coordinating Center will financially support specialty consultations, specialty services, and other study needs. It also is responsible for assisting the sites in developing and implementing common protocols, implementing quality assurance procedures, including site monitoring; developing data management systems and databases; and developing analytic capacity. The Data Coordinating Center will be responsible for the creation and integrity of study databases, analytic capacity, and support of and attendance at all network meetings.

### Data and Safety Monitoring Board

A Data and Safety Monitoring Board (DSMB) has been established by NICHD to monitor the safety of ongoing clinical trials. It also advises the NIH and the GN, including the Data Coordinating Center, on research design issues, data quality and analysis, and ethical and human subject issues. The DSMB members have expertise in clinical trial design and conduct; relevant basic, medical, and behavioral sciences research; and ethics and cultural competency issues, particularly with relevance to developing country populations. The Data Coordinating Center provides coordination, support, travel funding, and logistical arrangements for DSMB meetings and actions.

In addition, the network has established policies and procedures that govern its operations, including publication. These documents are under periodic review, and may be amended and supplemented at the discretion of the NICHD and the steering committee.

This funding opportunity will use the NIH Cooperative Research Project Grant (U01) award mechanism.

This funding opportunity uses the just-in-time budget concepts. It also uses the nonmodular budget format described in the PHS 398 application instructions (see http://grants.nih.gov/grants/funding/phs398/phs398.html). A detailed categorical budget for the “Initial Budget Period” and the “Entire Proposed Period of Support” is to be submitted with the application.

The NIH U01 is a cooperative agreement award mechanism. In the cooperative agreement mechanism, the PI retains the primary responsibility and dominant role for planning, directing, and executing the proposed project, with NIH staff being substantially involved as a partner with the PI, as described under the Section VI. 2. Administrative Requirements, “Cooperative Agreement Terms and Conditions of Award.”

The PHS 398 application instructions are available at http://grants.nih.gov/grants/funding/phs398/phs398.html in an interactive format. Applicants must use the currently approved version of the PHS 398. For further assistance contact GrantsInfo, 301-435-0714 (telecommunications for the hearing impaired: TTY 301-451-0088) or by e-mail: GrantsInfo@nih.gov.

Applications must be prepared using the most current PHS 398 research grant application instructions and forms. Applications must have a Dunn and Bradstreet (D&B) Data Universal Numbering System (DUNS) number as the universal identifier when applying for Federal grants or cooperative agreements. The D&B number can be obtained by calling 866-705-5711 or through the web site at http://www.dnb.com/us/. The D&B number should be entered on line 11 of the face page of the PHS 398 form.

The letter of intent date for this RFA is 26 June 2007, with the application receipt date 27 July 2007. The complete version of this RFA is available at http://grants.nih.gov/grants/guide/pa-files/RFA-HD-07-016.html.

Contact: Linda L. Wright, Center for Research for Mothers and Children, National Institute of Child Health and Human Development, 6100 Executive Boulevard, Room 4B05, MSC 7510, Bethesda, MD 20892-7510 USA, Rockville, MD 20852 USA (for express/courier service), 301-402-0830, fax: 301-480-7773, e-mail: wrightl@mail.nih.gov.

Reference: RFA-HD-07-016.

